# Association of DSC3 mRNA Down-Regulation in Prostate Cancer with Promoter Hypermethylation and Poor Prognosis

**DOI:** 10.1371/journal.pone.0092815

**Published:** 2014-03-24

**Authors:** Jincheng Pan, Yu Chen, Chengqiang Mo, Daohu Wang, Junxing Chen, Xiaopeng Mao, Shengjie Guo, Jintao Zhuang, Shaopeng Qiu

**Affiliations:** 1 Department of Urology, The First Affiliated Hospital, Sun Yat-Sen University, Guangzhou, China; 2 Department of Urology, Sun Yat-Sen University Cancer Center, Guangzhou, China; Institut national de la santé et de la recherche médicale, France

## Abstract

**Background:**

Desmocollin 3 (DSC3), a member of the cadherin gene superfamily, is associated with pathogenesis of some cancers, but its role in prostate cancer (PCa) remains largely unknown.

**Methods:**

DSC3 gene expression level in available PCa microarray dataset was examined using the Oncomine database. DSC3 transcript expression in prostate cell line panel and an independent tissue cohort (n = 52) was estimated by quantitative PCR (Q-PCR). Epigenetic status of DSC3 gene promoter in PCa was investigated by uploading three dataset (ENCODE Infinium 450K array data and two methylation sequencing) in UCSC genome browser. While pyrosequencing analysis measured promoter DNA methylation, Q-PCR estimates were obtained for DSC3 transcript re-expression after 5-Aza-deoxycytidine (5-Aza) treatment. Clinical relevance of DSC3 expression was studied by Kaplan-Meier survival analysis. Finally, functional studies monitoring cell proliferation, migration and invasion were performed in prostate cell lines after siRNA mediated DSC3 knockdown or following 5-Aza induced re-expression. EMT markers Vimentin and E-cadherin expression was measured by Western Blot.

**Results:**

Microarray data analyses revealed a significant decrease in DSC3 transcript expression in PCa, compared to benign samples. Q-PCR analysis of an independent cohort revealed DSC3 transcript down-regulation, both in PCa cell lines and tumor tissues but not in their benign counterpart. Examination of available NGS and Infinium data identified a role for epigenetic regulation DSC3 mRNA reduction in PCa. Pyrosequencing confirmed the increased DSC3 promoter methylation in cancer cell lines and restoration of transcript expression upon 5-Aza treatment further corroborated this epigenetic silencing mechanism. Importantly Kaplan-Meier analysis of an outcome cohort showed an association between loss of DSC3 expression and significantly increased risk of biochemical recurrence. Functional studies indicate a role for epithelial–mesenchymal transition in DSC3 regulated cell migration/invasion.

**Conclusion:**

Taken together, our data suggests that DNA methylation contributes to down-regulation of DSC3 in prostate cancer, and loss of DSC3 predicts poor clinical outcome.

## Introduction

Prostate cancer is the second most common cancer among men worldwide and more than 900,000 men are diagnosed with prostate cancer each year [1]. Although PSA is clinically used as a screening test with a sensitivity of 80%, its usefulness is limited due to its low specificity (20%), which may lead to unnecessary biopsies and overtreatment [2]. Despite the large effort in searching for biomarkers, prostate cancer clinical management still remains a challenge, due to suboptimal performance of existing diagnostic and prognostic tools[3].

Studies that identify and characterize cancer specific aberrant molecular events also reveal candidates with potential biomarker utility[4]. Differential DNA methylation is common in prostate cancer that results in hypermethylation at the promoter of tumor suppressor genes, hypomethylation at the promoters of oncogenes, and global hypomethylation leading to genomic instability at later stages of prostate cancer [5], [6]. Some of the commonly reported hypermethylated genes in prostate cancer include GSTP1, RASSF1A, and APC, which have aided in prostate cancer diagnosis and prognosis [7]. Prostate cancer is a heterogeneous disease, the number of hypermethylated genes rises as prostate cancer progresses [8], [9]. Importantly molecular events in PCa, such as gene fusions have been recently shown to impart differential DNA methylation patterns [5], [9]. This implies that PCa disease heterogeneity may be better reflected by differential epigenetic signatures. Thus, characterization of differentially methylated regions (DMRs) has a high probability to yield potential biomarkers with clinical use for prostate cancer.

Members of the desmosomal cadherin family, including desmocollins and desmogleins, are found primarily in epithelial cells where they constitute the adhesive proteins of the desmosome cell-cell junction and are required for cell adhesion and desmosome formation [10], [11]. Impaired desmosomal protein function is associated with multiple diseases [12]. One of the interesting functions is their ability to inhibit cell motility, a phenomenon of importance in cancer [10]. Desmocollin 3 (DSC3), a member of cadherin superfamily, contributes to desmosome mediated cell-cell adhesion[11]. Recent studies have observed loss of DSC3 in several cancer types, such as lymph node metastases of oral squamous cell carcinoma, breast cancer and colorectal cancer, where the decreased levels associated with cancer progression were regulated by epigenetic modification[13], [14], [15]. However, little is known about the expression and regulatory mechanisms of DSC3 in prostate cancer.

In this study, we analyzed the expression and methylation patterns of DSC3 in prostate cancer, and more importantly explored the functional role of DSC3 and its potential clinical prediction value.

## Materials and Methods

### Cell lines and Tissue samples

All prostate cell lines obtained from the American Type Culture Collection and the prostate primary cells PrEC purchased from Lonza were grown at 37°C in a 5% CO2 cell culture incubator. The prostate cancer cell lines were maintained in DMEM (Invitrogen) (Du145) and RPMI (LNCaP, 22RV1) supplemented with 10% fetal bovine serum (FBS). The normal prostate cell line RWPE was maintained in KSF media (Invitrogen) plus 10 ng/mL EGF (Sigma) and bovine pituitary extract (BPE) and the primary prostate cells PrEC was cultured in PrEGM media (Lonza). Prostate tissue samples including benign prostate (n = 18), prostate cancer (n = 34) were obtained from the First Affiliated Hospital of the Sun Yat-Sen University (Guangzhou, China). The tissue specimens were flash-frozen in liquid nitrogen at time of collection and stored at −80°C until RNA extraction. Informed written consent was obtained from all patients to allow the use of samples and clinical data for investigation. This study involving human subjects was approved by the Ethics Council of the Sun Yat-Sen University.

### RNA extraction and Quantitative RT–PCR

Total RNA isolated using TRIzol (Invitrogen) was utilized in cDNA synthesis (Superscript III, Invitrogen) in the presence of random primers (Invitrogen). Quantitative Real-time PCR (Q-PCR) was performed using Power SYBR Green MasterMix (Applied Biosystems) on an Applied Biosystems 7900HT Real-Time PCR System. All oligonucleotide primers were obtained from Invitrogen and are listed in **Table S1**. GAPDH amplification was used as internal control. The mRNA expression level was determined as previously described, using the 2^−ΔΔCT^ method [16].

### ENCODE dataset and Methylplex NGS sequencing data Analysis

The Infinium 450K Bead Array and Reduced Representation Bisulfite Sequencing DNA methylation data from ENCODE consortium available as user tracks in UCSC genome browser were utilized in this study[17]. The Methylplex NGS sequencing data custom tracks of benign cell line PrEC and PCa cell line LNCaP, prostate normal, benign adjacent, localized PCa, and metastatic PCa tissues from a recently published study by Kim et al. (Genome Res 2011) were also uploaded as tracks into UCSC genome browser and visualized[9]. This deep-sequencing data from a previously published dataset is deposited in NCBI GEO under accession number:GSE27619.The DSC3 genomic region “Chr18:28,570,052-28,622,781” (Hg19) (including both the promoter and coding region) was inspected for differential DNA methylation.

### Genomic DNA isolation, Bisulfite conversion and Pyrosequencing

The genomic DNA extracted with QIAamp DNA Mini Kit (Qiagen) was bisulfite converted using EZ DNA methylation gold kit (Zymo Research) and used as template for PCR reactions. DSC3 methylation was estimated using the PyroMark DSC3 methylation assay (Qiagen) according to the manufacturer's instructions. Briefly, bisulfite converted DNA, DSC3 primers, and Hotstart Master Mix (Qiagen) were used in a PCR reaction to amplify DSC3 genomic regions interest from the sample. The amplification was obtained from 45 cycles of 30 sec at 94°C, 30 sec at 50°C, and 40 sec at 72°C, after an initial enzyme activation for 15 min at 95°C, and final elongation of 7 min at 72°C. The PCR products were captured on Streptavidin Sepharose beads (GE Healthcare), denatured to produce single strands, washed, and annealed to sequencing primer, and the sequence was determined using the PyroMark Q24 system (Qiagen). The mean methylation of three individual positions is considered in this test. Primer sequences are show in **Table S1**. Methylation levels individually for each CpG site and its calculated average values are show in **Table S2.**

### 5-Aza-deoxycytidine Treatment

LNCaP cells seeded in 6-well plates were treated on the following day with indicated concentrations of 5-Aza-deoxycytidine (Sigma) or an equivalent volume of vehicle (DMSO) for five consecutive days. The growth medium containing either 5-Aza-deoxycytidine or vehicle was replaced every 24 hours. At the end of treatment period, total RNA was isolated to evaluate the indicated transcript expression by Real Time RT-PCR.

### Western Blot Analysis

Protein from cell lysates was isolated, and protein concentrations were determined by BCA kit. 20 μg of protein were separated by SDS-PAGE and transferred onto PVDF membrane (GE Healthcare). The membrane was incubated for half an hour in blocking buffer containing 5% milk followed by incubation overnight at 4°C with the primary antibodies, that include E-Cadherin antibody (1∶1000,3195S, Cell Signaling); Vimentin antibody (1∶1000,5741S, Cell Signaling) or GAPDH antibody (1∶2000,5174, Cell Signaling). Following several washes with Tris Buffered Saline containing Tween-20 (TBS-T), the blot was incubated with horseradish peroxidase-conjugated secondary antibody. The signals were visualized by enhanced chemiluminescence system as per manufacturer's protocol (GE Healthcare).

### RNA interference

Cells were plated in 6-well plates at a desired numbers and transfected with DSC3 siRNA (Dharmacon) or non-targeting siRNA twice. Transfections were performed with OptiMEM (Invitrogen) and oligofectamine (Invitrogen) according to standard protocols. Twenty four hours after transfection, cells were trypsinized and plated in triplicate at 8,000 cells per well in 24-well plates. Knockdown efficiency was determined by Q-PCR.

### Cell Proliferation/Migration/Invasion Assay

Cell proliferation assay was conducted using the WST-1 Kit according to the manufacturer's instruction (Clontech Laboratories). For Migration/Invasion, cells were transfected or treated. After 24 hours, cells were seeded on 8 μm inserts (BD Falcon) coated with Matrigel (for invasion assays) or uncoated (for motility assays) in a 24-well culture plates. Fetal bovine serum was added to the lower chamber as a chemo attractant. After 72 hours, non-invading cells were gently removed by cotton swab. Invasive cells on the lower side of the chamber were stained with crystal violet and air-dried. Relative invasion was quantitated by solubilization of crystal violet dye and measurement of absorbance at 560 nm.

### Oncomine Expression and Outcome Analysis

DSC3 expression from independent published microarray studies was extracted from Oncomine database. The datasets can also be obtained from NCBI Gene Expression Omnibus (GEO) under accession numbers, GSE35988 (Grasso et al.,); PMID: 19737960 (Arredouani et al.,); GSE3325 (Varambally et al.,); GSE3933 (Lapointe et al.,); and GSE21032 (Taylor et al.,). For Kaplan-Meier analysis of the Taylor et al., (Cancer Cell 2010) dataset[18], biochemical recurrence was defined as a 0.2 ng/ml increase in PSA or recurrence of disease after prostatectomy, such as development of metastatic cancer, if biochemical recurrence information was not available. DSC3 expression values were categorized into low and high groups according to the median cutoff.

### Statistical Analysis

All statistical analysis was carried out using GraphPad Prism software program. For quantitative data, groups were reported as mean ± SEM and compared using the unpaired Student's t-test or one-way ANOVA test. For Kaplan-Meier survival analysis, log-rank test was used. Statistical significance was established at P<0.05.

## Results

### DSC3 expression is suppressed in prostate cancer

**Figure 1:**
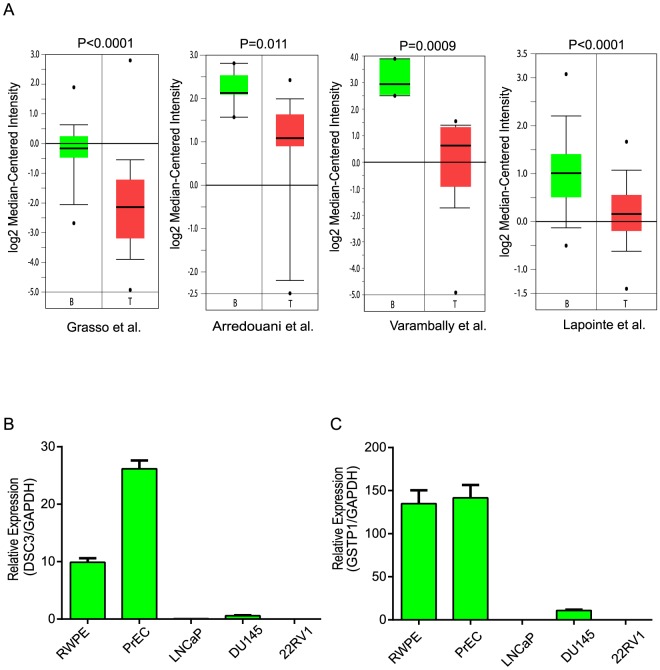
DSC3 Expression is Downregulated in Prostate Cancer. (A) Boxplots representation of DSC3 mRNA expression in four independent prostate cancer microarray datasets, B = Benign, T = Tumor. First author and statistical significance are indicated. (B) Q-PCR analysis of DSC3 and (C) GSTP1 expression in a panel of prostate cell lines; PrEC-prostate normal epithelial cells, RWPE-benign prostate cell line, LNCaP, Du145, 22RV1 are metastatic prostate cancer cell lines.

To explore the expression of DSC3 transcript in prostate cancer and benign tissues, we used the Oncomine tool to analyze several published microarray gene expression studies. Our analysis revealed a strong reduction in DSC3 mRNA expression in prostate cancer tissues as compared to benign specimens. **Figure 1A** depicts the levels of DSC3 transcripts across four independent published microarray studies [19], [20], [21], [22]. To experimentally corroborate this observation, we tested a panel of prostate cell lines including LNCaP, DU145, 22RV1, benign cells namely RWPE and PrEC, by Q-PCR. In a pattern consistent with microarray data the benign cells showed higher transcripts expression when compared with cancer cell lines (**Figure 1B**). Similar expression pattern was observed for GSTP1, a well-known methylated gene in prostate cancer which we used as a positive control (**Figure 1C**).

### DSC3 down-regulation in PCa is mediated by promoter methylation

**Figure 2:**
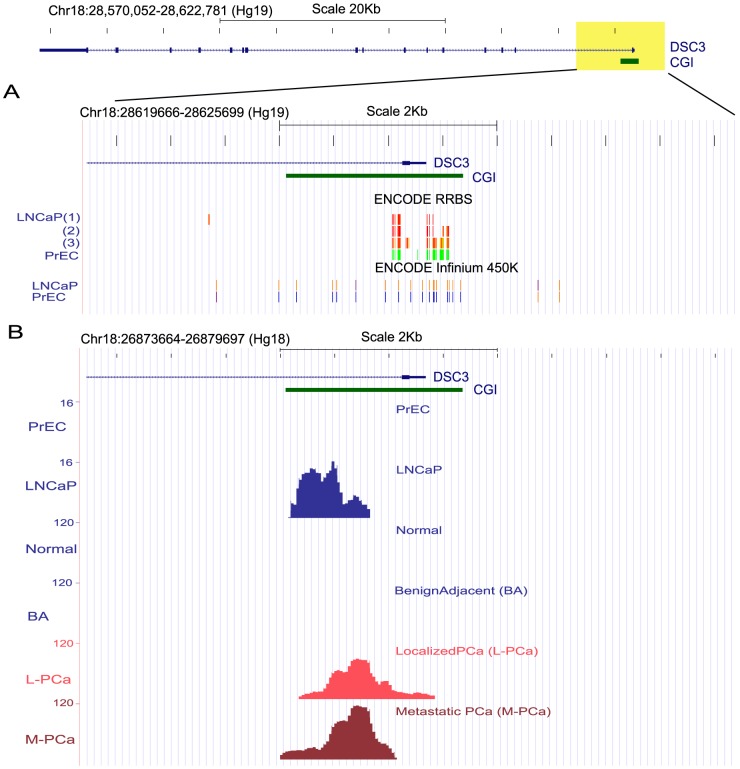
DSC3 Promoter is Hypermethylated in Prostate Cancer. (A) Top panel depicts the UCSC genome browser representation of the genomic organization of DSC3 gene present in human chromosome 18 (28570052-28622781), where exons are indicated by solid boxes and introns by the blue line. The CpG island (CGI) associated with the DSC3 gene promoter is represented as horizontal green solid bar. DNA methylation status of the regions shaded in yellow (+/−3 kb from transcription start site) is presented below. ENCODE Infinium 450K Bead Arrays and Reduced Representation Bisulfite Sequencing datasets (RRBS) showed that DSC3 gene promoter is hypermethylated in LNCaP as compared to the benign cell line PrEC. The CG positions assayed by these methods are represented as vertical bars colored according to their methylation status. ENCODE RRBS, red (100%), yellow (50%), green (0%) are methylated; ENCODE Infinium 450K, orange  =  methylated (score ≥600), bright blue  =  unmethylated (0< score ≤200). LNCaP(1):LNCaP cells treated with androgen; LNCaP(2):LNCaP data from Duke University; LNCaP(3):LNCaP data from University of Washington. (B) Representation of MethylPlex NGS sequencing data (PrEC, LNCaP, prostate normal, benign adjacent, localized PCa and metastatic PCa tissues) for DSC3. Methylated regions observed in the corresponding sample groups are depicted as peaks and numbers in the y-axis denote the peak heights.

**Figure 3:**
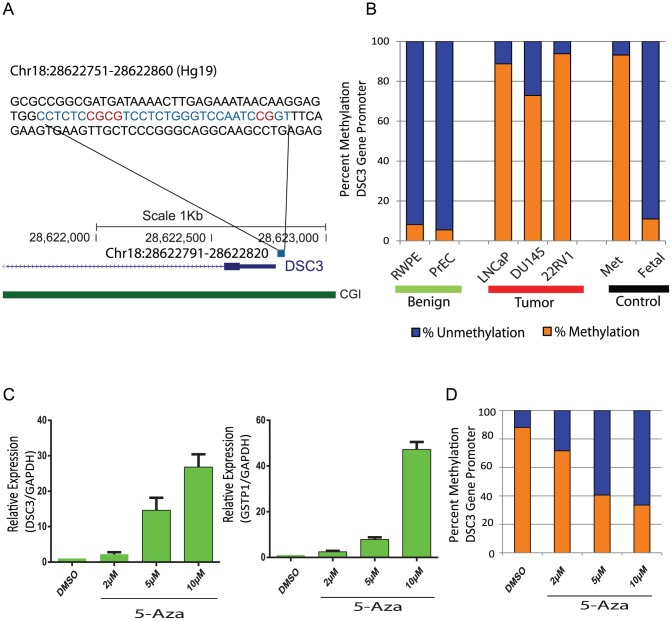
Hypermethylation of DSC3 Promoter in PCa Cell Lines. (A) Sequence of the DSC3 promoter (Chr18:28622751-28622860) and the region analyzed by pyrosequencing are given in blue (Chr18:28622791-28622820) and the CG positions monitored within are indicated in red. CGI, CpG island. (B) Bar plot depicts the average of the percent methylation in the three CG positions in prostate cell lines. Blue-percent unmethylation; Orange-percent methylation; Bi-sulfite converted universally methylated gDNA and fetal DNA were used as positive and negative controls respectively. (C) DSC3 expression as assessed by Q-PCR in LNCaP cells treated with 5-Aza-deoxycytidine (5-Aza), GSTP1 was included as a positive control. (D) Bar plot depicts the average of the percent methylation in the three CG positions in LNCaP treated with 5-Aza.

A detailed examination of DSC3 genomic organization using the UCSC genome browser revealed the presence of a CpG island in DSC3 gene regulatory region “Chr18:28,570,052-28,622,781” (Hg19) surrounding the transcription start site. Analysis of DNA methylation in this region in ENCODE Methyl 450K Bead Arrays and Reduced Representation Bisulfite Sequencing (RRBS) datasets[17] showed that the DSC3 gene promoter is hypermethylated in LNCaP as compared to the benign cell line PrEC (**Figure 2A**). In addition, analysis of an independent prostate cancer DNA methylation dataset[9] generated by Methylplex Next Generation Sequencing (M-NGS) also revealed DSC3 promoter methylation in LNCaP and not in PrEC. In addition this data also showed increased methylation in localized and metastatic prostate cancer samples and not in benign or normal prostate specimens (**Figure 2B**). Both the cell line data from ENCODE[17] and cell line/tissue dataset from the M-NGS study by Kim et al.[9], revealed DNA methylation was in a highly overlapping region within the DSC3 promoter. The base pair resolution of these datasets, allowed us to accurately home in on the chromosomal region targeted by DNA methylation. Using the above information we next experimentally investigated the chromosomal region in DSC3 promoter for methylation change by pyrosequencing analysis in a panel of prostate cell lines. Supporting the trend observed with the publically available data, pyrosequencing analysis revealed frequent hypermethylation of DSC3 promoter in cancer cell lines (3/3) compared with benign cell lines (0/2) (10% methylation as a cutoff) (**Figure 3B**). In order to test whether DSC3 is epigenetically silenced by methylation in prostate cancer, we treated LNCaP cells with a DNA methylation inhibitor, 5-Aza-deoxycytidine (5-Aza). Transcript expression analyses by Q-PCR of both DSC3 and GSTP1, the latter used as a positive control showed induction of both these mRNAs upon 5-Aza drug treatment, and pyrosequencing showed that a reduction in DNA methylation of the DSC3 promoter region upon 5-Aza treatment (**Figure 3C&D**). This data indicates that DSC3 transcription similar to GSTP1, is epigenetically silenced due to DNA hypermethylation in prostate cancer. Taken together these observations suggest that DSC3 gene promoter is subject to frequent methylation in both prostate cancer tissues and cell lines. Our experiments in cell line model also lent support to the notion that DSC3 gene transcription is regulated by CpG island hypermethylation in prostate cancer.

### DSC3 is down-regulated in PCa and predicts poor clinical outcome

**Figure 4:**
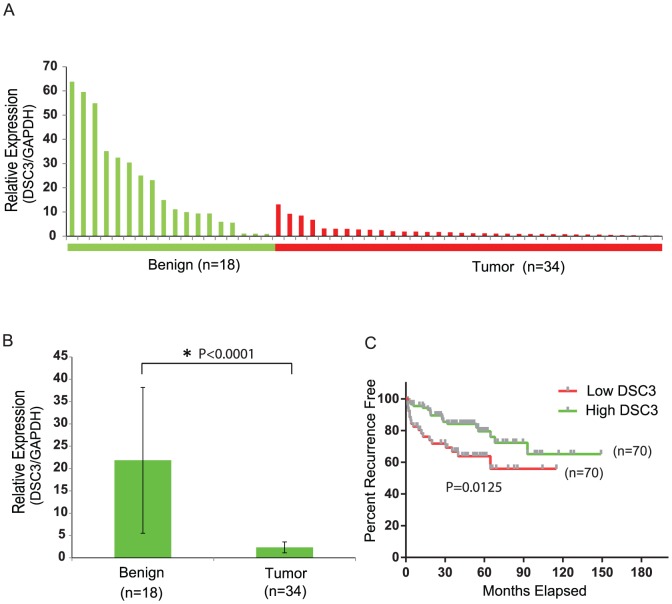
DSC3 is mRNA is Down-regulated in an Independent PCa Cohort and its Expression Correlates with Biochemical Recurrence. (A) Q-PCR for DSC3 transcript in a cohort of benign prostate (n = 18) and prostate cancer (n = 34) tissues. (B) A summary histogram of the expression values of DSC3 in prostate benign and tumor samples. (C) Kaplan-Meier analysis showed that low DSC3 group patients had a significantly increased risk of recurrence than the high DSC3 group patients (P = 0.0125).

To explore the expression of DSC3 in prostate cancer tissues, we performed Q-PCR on a panel of prostate tissues including benign prostate (n = 18), prostate cancer (n = 34). DSC3 expression was remarkably decreased in prostate cancer (2.4±1.2) compared to benign tissues (21.8±16.3) (**Figures 4A&B**). Approximately 25–40% of patients treated by radical prostatectomy for clinically localized prostate cancer will experience disease recurrence, initially indicated by an increase in the serum level of PSA (biochemical recurrence)[23]. Thus, we next sought to determine if DSC3 inactivation status was associated with biochemical recurrence after surgical resection. Towards this we examined the Taylor et al. (Cancer Cell 2010) gene expression dataset [18], which characterized tumors from 140 patients who have recurrence information (with 36 recurrences). The samples were separated into high and low DSC3 groups based on DSC3 median expression value. Kaplan-Meier analysis revealed that low DSC3 group patients had a significantly higher risk of recurrence than the high DSC3 group patients (Hazard ratio: 2.352, 95% CI: 1.198–4.446, log rank P = 0.0125) (**Figure 4C**). This suggests that DSC3 transcript loss in prostate cancer can predict poor clinical outcome. Collectively, these data suggests DSC3, a member of cadherin superfamily might be playing an important role in prostate cancer progression leading to poor clinical outcome.

### Effects of DSC3 on the migration/invasion potential of prostate cells

**Figure 5:**
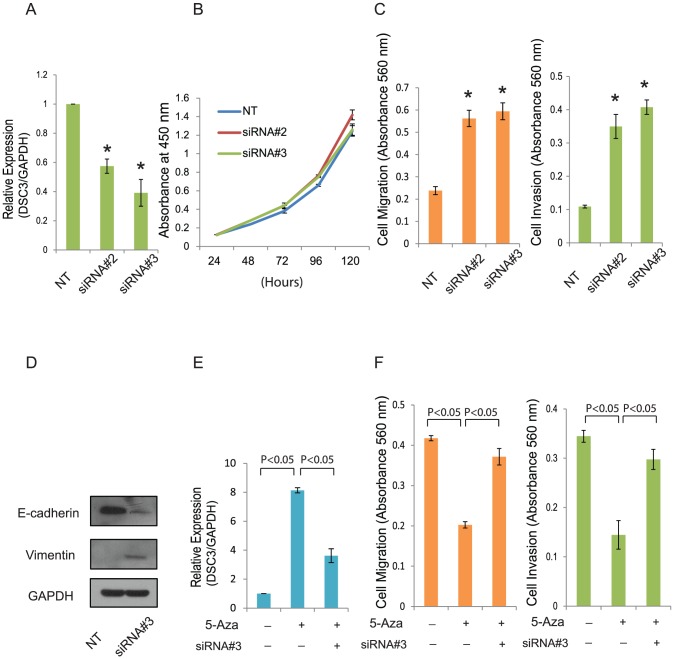
Effect of DSC3 on the Migration/Invasion Potential of Prostate Cells. (A) DSC3 knockdown by siRNA in benign prostate cell line RWPE as detected by Q-PCR.*indicates values that are significantly different than the Non-Target group (NT). siRNA#2 and siRNA#3 are two individual DSC3 siRNA. (B) Effect of DSC3 knockdown on proliferation in RWPE cells by WST-1. (C) Effect of DSC3 knockdown on migration through Transwell and invasion through Matrigel in RWPE cells. Migrated and invaded cells were stained with crystal violet, solubilized and quantitated by measuring absorbance at 560 nm.*indicates values that are significantly different than the Non-Target group (NT). (D) E-cadherin and Vimentin expression assessed by Western blot analysis in RWPE cell treated with Non-Target siRNA or DSC3 siRNA. (E) Q-PCR for DSC3 using total RNA extracted from PCa cell line Du145 treated with 5-Aza or DSC3 siRNA. (F) The migration and invasion potential of the untreated and 5-Aza treated Du145 were assessed by Transwell or Matrigel assay. Migrated and invaded cells were stained with crystal violet, solubilized and quantitated by measuring absorbance at 560 nm.

DSC3 is one of the adhesive proteins of the desmosome cell-cell junction and is required for cell adhesion. To study the role of DSC3 in prostate, we transfected benign RWPE cells with either control or DSC3 siRNA and performed proliferation, migration and invasion assays. Transient knockdown of DSC3 in RWPE resulted in increased cell migration and invasion (**Figure 5C**), while no significant effect was observed on cell proliferation (**Figure 5B**). To understand the mechanism of DSC3 regulated migration and invasion, we tested the expression of epithelial–mesenchymal transition (EMT) marker E-cadherin and Vimentin in RWPE treated with either control or DSC3 siRNA. Interestingly, knockdown DSC3 in RWPE increased Vimentin expression while inhibiting E-cadherin expression (**Figure 5D**). This suggested that DSC3 might regulate prostate cell line migration/invasion potential by inducing EMT. To determine the effect of DSC3 re-expression on the function of prostate cells, the migration and invasive potential of control and 5-Aza treated DU145 cells were assessed. Induction of DSC3 expression upon 5-Aza treatment of DU145 cells resulted in a significant reduction of the migration/invasion potential of these cells. Further treatment of these cells with DSC3 siRNA, partially restored the migration/invasion potential (**Figure 5F**). These results render convincing evidence that the decreased cancer cell migration/invasion after treatment with 5-Aza is mainly due to the induction of DSC3 expression.

## Discussion

Desmocollin 3 (DSC3) was found to be frequently down-regulated in several cancers such as breast, oral, colorectal and lung cancer, and inactivation of DSC3 in these cancers was caused by promoter hypermethylation[13], [14], [15], [24]. However, little is known about the role of DSC3 in prostate cancer. Towards this we first compared DSC3 expression in prostate cancer from published microarray gene expression studies using the Oncomine tool. Four independent PCa datasets revealed that DSC3 was significantly reduced in prostate cancer tissues compared with benign samples. Our analysis also showed consistent downregulation of DSC3 mRNA in cancer cell lines. Next, we explored various publically available dataset for the methylation status of DSC3 gene promoter in prostate cancer. Multiple lines of evidence based on analysis using independent platforms such as Infinium Methylation 450K Bead Arrays (ENCODE), Reduced Representation Bisulfite Sequencing (ENCODE) which characterized LNCaP and PrEC cells, along with MethylPlex NGS sequencing study that characterized LNCaP, PrEC and prostate cancer clinical specimens supported differential methylation of DSC3 gene promoter in prostate cancer. We also demonstrated that DSC3 gene is epigenetically silenced in prostate cancer, as treatment of LNCaP cells with the de-methylating agent 5-Aza, induced DSC3 transcript expression. Our present study thus demonstrates that DSC3 expression is frequently lost in prostate cancer due to promoter hypermethylation similar to previous reports in other solid tumors [15], [24]. Importantly DSC3 expression in prostate cancer clinical specimens was able to predict poor clinical outcome when analyzed for biochemical recurrence.

A functional study conducted thus far has identified DSC3 as a potential tumor suppressor gene, stable transfection of a DSC3 expression vector in lung cancer cell lines showed that ectopic expression of DSC3 inhibited cell proliferation, anchorage-independent growth, migration, as well as invasion [24]. Interestingly we observed an increase in cell migration and invasion of RWPE prostate cells when we knocked down DSC3 gene using two specific independent siRNAs and not the control non-target siRNA. The siRNA mediated silencing of DSC3 did not show any effect on cell proliferation. In addition, knockdown of DSC3 while inducing Vimentin protein expression, decreased the levels of E-Cadherin, probably suggesting an EMT like phenotype when DSC3 gene is down-regulated. While the regulatory mechanisms controlling DSC3 expression are not fully understood, DSC3 is a TP53 response gene and addition of wild-type TP53 was found to be sufficient to induce expression of DSC3 in breast cancer [15], [25]. Hence loss of TP53 function through somatic mutation, which is frequently observed in advanced prostate cancer, may result in hypermethylation of its target genes. A similar relationship was observed with ERG expression and methylation status of its target gene namely TDRD1 in prostate cancer [26], [27]. However, more studies are required to explore the mechanisms regulating DSC3 in prostate cancer.

To clarify the expression of DSC3 in prostate cancer tissue cohort from a Chinese population, we performed Q-PCR on prostate tissues. DSC3 expression was significantly and strongly decreased in prostate cancer compared to benign tissues. Given its inactivation in PCa cell lines and tissues, we want to assess whether DSC3 expression is associated with poor clinical outcome. In our analysis we observed a correlation between down-regulation of DSC3 and significantly higher risk of biochemical recurrence. Aberrant DNA methylation of gene promoters is characteristic of cancer cells, and several genes are epigenetically altered in a cancer specific manner [28]. In prostate cancer patients, correlations between specific gene promoter hypermethylation and Gleason score, pathologic stage or tumor recurrence is well known[29]. GSTP1 gene promoter methylation is widely characterized by several independent groups and is found to be have diagnostic value as a biomarker in prostate cancer patient tissue or body fluid aided in non-invasive detection[30]. Hypermethylation of DSC3 was previously reported as a marker to predict clinical outcome in colorectal and lung cancer [15], [24]. Thus, more studies on large prostate cancer tissue cohorts as well as body fluids are needed to further evaluate DSC3 as a methylation biomarker. Such studies will comprehensively establish the association between DSC3 promoter methylation and prostate cancer clinical characteristics.

## Conclusion

In summary, DSC3 is down regulated in prostate cancer by DNA hypermethylation. This is the first study, to report a detailed analysis of DSC3 mRNA expression and gene promoter methylation in prostate cancer. Analysis of the expression of DSC3 might be useful to predict clinical outcomes in prostate cancer patients. Future studies are necessary to expand these observations and investigate specifically the diagnostic/therapeutic potential of DSC3 in prostate cancer.

## Supporting Information

Table S1 Summary of Sequences of primers.
(XLSX)

Table S2 Summary of Pyrosequencing of three CG position in prostate cell lines.
(XLSX)
